# Uterine preservation for advanced pelvic organ prolapse repair: Anatomical results and patient satisfaction

**DOI:** 10.1590/S1677-5538.IBJU.2015.0656

**Published:** 2016

**Authors:** Keshet Fink, Inbar Ben Shachar, Naama Marcus Braun

**Affiliations:** 1 Bar Ilan University - Faculty of health, Safed, Israel; 2 Ziv Medical Center - Ob/Gyn, Safed, Israel

**Keywords:** Pelvic Organ Prolapse, Vagina, Autonomic Nervous System, Uterus

## Abstract

**Objective:**

The aims of the current study were to evaluate outcomes and patient satisfaction in cases of uterine prolapse treated with vaginal mesh, while preserving the uterus.

**Materials and Methods:**

This is a retrospective cohort study that included all patients operated for prolapse repair with trocar-less vaginal mesh while preserving the uterus between October 2010 and March 2013. Data included: patients pre-and post-operative symptoms, POP-Q and operative complications. Success was defined as prolapse < than stage 2. A telephone survey questionnaire was used to evaluate patient's satisfaction.

**Results:**

Sixty-six patients with pelvic organ prolapse stage 3, including uterine pro-lapse of at least stage 2 (mean point C at+1.4 (range+8-(-1)) were included. Mean follow-up was 22 months. Success rate of the vaginal mesh procedure aimed to repair uterine prolapse was 92% (61/66), with mean point C at −6.7 (range (-1) - (-9)). No major intra-or post-operative complication occurred. A telephone survey questionnaire was conducted post-operatively 28 months on average. Ninety-eight percent of women were satisfied with the decision to preserve their uterus. Eighteen patients (34%) received prior consultation elsewhere for hysterectomy due to their prolapse, and decided to have the operation at our center in order to preserve the uterus.

**Conclusions:**

Uterine preservation with vaginal mesh was found to be a safe and effective treatment, even in cases with advanced uterine prolapse. Most patients prefer to keep their uterus. Uterus preservation options should be discussed with every patient before surgery for pelvic organ prolapse.

## INTRODUCTION

The prevalence of symptomatic pelvic organ prolapse (POP) is difficult to estimate because of lack of standardized methods to evaluate symptomatic prolapse, and lack of data concerning the proportion of women with POP who do not seek medical aid (1). Nevertheless, it is possible to estimate the prevalence of symptomatic POP by the number of patients who choose to undergo surgical repair. It has been estimated that the lifetime risk for American or Australian women to have an operation for POP is 11% and 19% respectively (2, 3). Among the prolapsed compartments, the anterior compartment is the most common prolapse, three times more common than posterior compartment and twice as common as apical prolapse (uterus or vaginal vault). But POP is dynamic and about two thirds of women with prolapse have genital prolapse of more than one compartment (4–6).

In the last decade, several authors have claimed that it is preferable to treat POP while preserving the uterus, even if future pregnancy is not desired and in the postmenopausal period. Advances in vaginal mesh surgery have resulted in new techniques for preserving the uterus (7, 8). At the same time, treatment of POP with synthetic mesh has become common (9). Some safety concerns for the use of grafts in POP repair have led the US Food and Drug Administration (FDA) to publish a safety notification in 2011, and subsequently guidelines for the use of vaginal meshes (10, 11).

There are several reasons for uterine preservation, apart from the early and late complications of hysterectomy. These include cultural beliefs, personal preferences, sexual identity, and reproductive preservation in young patients (12).

The EndoFast system (Allium-IBI, Israel) is a vaginal mesh kit for single-incision POP repair (13). The posterior kit is designed for apical pro-lapse repair and the arms of the mesh are fixated to the sacrospinous ligament with a metallic spider fastener. The body of the posterior mesh can be used or removed depending on concomitant advanced posterior compartment prolapse.

The aims of the current study were to evaluate the outcome of uterine preservation with the EndoFast system in cases of advanced POP and uterine prolapse and to assess patient satisfaction with the decision to preserve the uterus and with the operative procedure in general.

## MATERIALS AND METHODS

This is a retrospective cohort study, including a telephone questioner. The study was approved by the hospital ethics committee. We included all patients who underwent POP repair with uterine preservation at Ziv Medical Center between October 2010 and March 2013, using the EndoFast system (Allium-IBI, Israel).

At Ziv Medical Center, the POP repair protocol does not include routine hysterectomy. Patients with advanced POP are admitted for surgical repair with synthetic mesh. The patients undergo an evaluation for pre-malignant and malignant disease of the uterus, including personal and family history of cancer. If there is no contraindication, patients are advised to preserve the uterus.

All patients included in the study had at least symptomatic uterine prolapse stage 2. All patients included underwent apical repair with the posterior kit of the EndoFast system, with or without additional anterior or posterior repair, while preserving the uterus. Data were collected from computerized archive files. Data included demographic features such as age, parity, BMI, medical history, previous gynecological operations, and family history of POP. Pre-operative evaluation included patient's symptoms for bulge, pain, dyspareunia, voiding dysfunction, urgency, urinary incontinence, nocturia, constipation, dysphasia, POP-Q examination, and stress urinary incontinence (SUI) (obvious or occult). The stage of the prolapse was determined based on the most pro-lapsed compartment. Data regarding the operation included intra-operative and immediate post-operative complications. Each patient was examined after the surgery at 6 weeks, 6 months and 1 year with continuous follow-up once a year. At each visit, data were collected for patient's symptoms, POP-Q examination, and complications such as pain, de novo dyspareunia, mesh erosion, de novo urgency, voiding dysfunction, and de novo SUI. Success was defined as no bulge symptoms and prolapse < than stage 2. Additionally, a phone interview was conducted to estimate patient satisfaction with the consultation they had before the surgery, with the decision to preserve the uterus, and with the entire process (Appendix 1). Validated questionnaires for this subject are lacking. We formulated the questionnaire in a manner that does not hint at any possible advantage or disadvantage of the process.

## RESULTS

**Figure 1:**
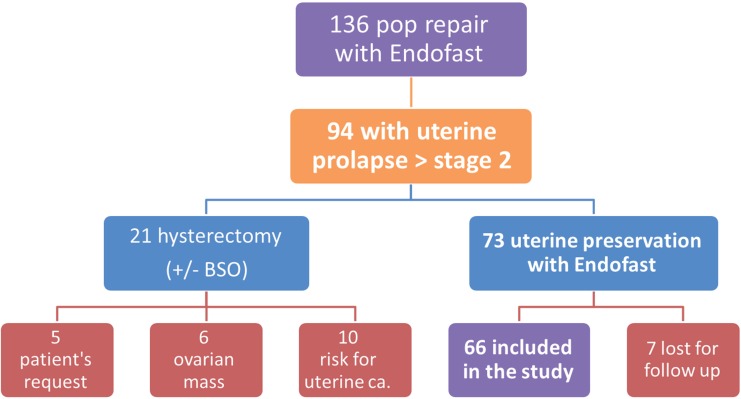
Patient selection for the study. Risk for uterine cancer was consider as a patient with breast cancer, currently under tamoxifen treatment

During the study period, 136 patients with POP were treated with the EndoFast system while preserving the uterus and 66 met our inclusion criteria and were included in the study (Figure-1). Fifty-one patients (77%) had additional anterior mesh placement for anterior compartment repair. Mean age was 61 (range: 43-82), mean parity was 4.31 (range: 1-12), mean BMI was 27.9 (range: 19.1-37.8), and mean follow-up at the clinic was 22 months (range: 6-42).

Before the surgery, 5, 57, and 4 patients had symptomatic POP stage 2, 3, and 4, respectively. All patients had at least uterine prolapse stage 2, with mean point C at +1.4 (range: + 8 -(-1)) (Table-1).

**Table 1:** Mean pre- and post-operative (at last visit) POP-Q.

	Pre-op	Post-op
**Ba**	1.4 (+10 - (-3))	-2.1 (+3 - (-3))
**Bp**	-1.6 (+7 - (-3))	-2.8 ((-2) - (-3))
**C**	1.4 (+8 - (-1))	-6.7 ((-1) - (-9))
**D**	-1 (+7 - (-6))	-8.2 (0 - (-12))

No intra-operative complications were reported. Immediate post-operative complications included one case of fever due to hematoma, which was self-resolved, and two cases of urinary tract infection. Long-term complications included (a) two cases of small erosions in the anterior mesh, of less than 5mm, which were treated locally with estrogen. (b) One case of metallic fastener removed from the rectum 2 months after the operation, without further sequel. Consistent pain or dyspareunia were not observed. Patient symptoms and functional results are shown in Table-2. All symptoms improved after the repair.

**Table 2:** Pre-operative symptoms and post-operative functional results (at last visit).

	SUI	De novo SUI	Dyspareunia	De novo dyspareunia	Urgency	De novo urgency
Pre-op	34		12		34	
Post-op	8	1	6	0	12	2

Operative success rate for uterine prolapse was 92% (61/66), with mean point C at - 6.7 (range (-1) - (-9)) and mean point D at −8.2 (range 0 - (-12)). Four women (6%) had recurrence of uterine prolapse within the first 6 months (> stage 1), but only 2 were symptomatic and required recurrent surgery. One patient had isolated elongation of uterine cervix without uterine prolapse. Pre-and post-operative POP-Q measurements are shown in Table-1.

Telephonic interviews were conducted on average 28 months after the surgery (Appendix 1). Fifty-three patients out of 66 (80%) were interviewed. When asked about the pre-operative consultations before the decision to preserve the uterus: 18 patients (34%) received prior consultation elsewhere for hysterectomy because of their pro-lapse, and decided to undergo the surgery at our center in order to preserve the uterus. Forty-eight patients (91%) reported the operation to have been successful, and 52 out of 53 patients (98%) were satisfied with the decision to preserve their uterus. In general, 49 patients (92%) were satisfied [17] or very satisfied [32] with the overall process.

## DISCUSSION

Hysterectomy is the second most frequently performed surgical procedure, after cesarean section, for US women. Approximately 400.000 hysterectomies are performed annually (14). Routine hysterectomy for uterine prolapse is no longer mandatory, and many recent studies support uterine preservation. The uterus can be preserved through vaginal route correction, with or without mesh, and is usually fixed to the sacrospinous ligament. The uterus can also be preserved using an abdominal or laparoscopic approach, such as sacrohysteropexy, which has produced good results (7, 8).

There are several medical reasons for preserving the uterus: (a) avoiding early and late complications of hysterectomy; (b) decreasing the rate of mesh erosion if a mesh is used at the time of hysterectomy (8); (c) reducing the cost of surgery with a shorter operation and hospitalization time (15); and (d) risk of vault prolapse, which is greater in women who had previous hysterectomy, especially after vaginal hysterectomy due to pro-lapse, as shown in several studies (16, 17). Other reasons for patient's desire to preserve the uterus include desire to sustain fertility, maintaining personal identity, cultural and religious considerations (18). Preservation of the uterus was shown to contribute positively to patient's self-esteem, body image, confidence, and sexuality (15).

In the past, several uterine preservation methods have been developed for selected young women suffering from uterine prolapse who desire to remain fertile. The Manchester procedure, mainly for cervical elongation, was developed already in the late 1890s. It is a good method for uterine preservation, but recurrence rate increases when the prolapse is more advanced. It is also associated with cervical stenosis, and therefore not recommended today for fertile women (7). Sacrospinous hysteropexy was first described by Richardson in 1989 (19). It involves sacrospinous fixation with suture or sutures, unilateral or bilateral. Several studies have demonstrated its success rate and pregnancy rate (7). In the 1950s, a large series in which the uterus was preserved in young women by suturing to the abdominal wall, demonstrated a high success rate, with non-negligible pregnancy rate after the surgery (20).

Since the introduction of the vaginal mesh, at the beginning of the current century, many series have demonstrated good results of its use in preserving the uterus (8). This concept has led to a new approach in which the uterus can be preserved not only for purposes of fertility, but in any prolapse. Women often ask to preserve the uterus, an option that should always be discussed before surgery. Previous studies that examined patient satisfaction with hysterectomy in non-malignant situations, such as heavy bleeding and prolapse, have found a high rate of satisfaction with the operation (21, 22). But this may be the result of the fact that in the past patients were given no choice, as the only option for POP repair was vaginal hysterectomy and native tissue repair; patient satisfaction, therefore, may have been due to the relief from symptoms. Studies evaluating women's preference before the operation are lacking. Frik et al. examined 220 patients evaluated for the presence of POP. Sixty percent stated that they would prefer preserving their uterus if a good alternative was available (23). Another study examined 213 patients who had POP and desired prolapse repair. 36% preferred uterine preservation as opposed to only 20% who chose hysterectomy, assuming similar outcomes in both procedures (18). In our study, we have found that 18 patients (34%) had pre-consultation for hysterectomy elsewhere and decided to undergo the surgery at our center in order to avoid hysterectomy.

To estimate the patient satisfaction with the overall process, we conducted a phone survey 28 months on average after the surgery. The survey showed that 91% of patients evaluated the results as successful. We were also able to evaluate patient satisfaction with the decision to preserve the uterus. We have found that 98% of patients were satisfied with the decision to preserve the uterus. Unfortunately, women are still being advised that hysterectomy is the only solution to their prolapse. In our study, one third of the women sought an alternative.

The limitations of this study include its retrospective nature and the use of telephone survey and not validated questionnaires. Moreover, our center protocol to avoid routine hysterectomy may cause bias discussion around patient satisfaction as one-third of the patients were seeking preservation pre-op. Large RCTs are required in order to overcome surgeons and patients bias so the results can be applicable to the population in general. At the same time, the study is strengthened due to the focus on patients with advanced uterine prolapse while excluding those with non significant uterine prolapse and by included a large population of patients with detailed pre-and post-operative physical evaluations and a long thorough follow-up. In addition, all patients were operated by the same surgeon (MN) in the same institution thus neutralizing inter-surgeons differences.

## CONCLUSIONS

Uterine preservation in patients with significant uterine prolapse undergoing POP repair with trocar-less vaginal mesh is safe and effective. Most patients in our study preferred to preserve their uterus even in their post-reproductive age and were satisfied with the operative results. Uterus preservation options should be discussed with every patient before surgery for POP.
